# Spontaneous development of myasthenia gravis and myositis following treatment with pembrolizumab: a case report

**DOI:** 10.1186/s12883-024-03684-2

**Published:** 2024-06-01

**Authors:** Benjamin Kiaei, Maaria Chaudhry, Sumona Banerjee, Jonathan Brewer, Yongzhen Chen, Farid Khasiyev, Miguel A. Guzman, Ghazala Hayat

**Affiliations:** 1 Department of Neurology, Saint Louis University Hospital, St. Louis, USA; 2 Department of Pathology, Saint Louis University Hospital, St. Louis, USA

**Keywords:** Myasthenia gravis, PD-1 inhibitor, Myositis, Keytruda, Pembrolizumab, Immune checkpoint inhibitor

## Abstract

**Background:**

Immune checkpoint inhibitors are a relatively new advancement in the world of cancer therapy. As such, their adverse effects have yet to be fully understood, with only recent literature documenting autoimmune phenomena secondary to their utilization. Specific immune checkpoint inhibitors have recently been linked with the development of myasthenia gravis, which is classically known to manifest spontaneously in patients. Given the relative rarity of this presentation, the risk of misdiagnosis and subsequent mortality and morbidity is concerning.

**Case presentation:**

We discuss the case of a 73-year-old male who presented with clinical symptoms of myasthenia gravis and myositis shortly after beginning treatment with Pembrolizumab. The diagnosis of myasthenia gravis was initially missed at an outside hospital, which delayed initiation of proper treatment.

**Conclusion:**

While the incidence of “*de-novo”* diseases secondary to immune checkpoint inhibitors might be increasing, guidelines regarding best treatment options do not yet exist, leaving many providers at a loss when faced with making clinical decisions surrounding patients with De novo myasthenia gravis. Thus, our goal is to underscore the importance of early recognition of this disease, and emphasize the need for a standard of care as immune checkpoint inhibitors usage becomes more prevalent.

## Background

Immune checkpoint inhibitors have been pivotal in the treatment of many forms of cancer. However, due to the relative novelty of these therapies, the breadth of their complications remains under-studied [1]. Checkpoint inhibitors target proteins such as CTLA-4 and PD-1 found in T-cells [2]. By preventing these proteins from binding their receptors, T-cells can remain active and further target cancer cells. Naturally, these checkpoint inhibitors activate a non-specific immune response which can have systemic adverse effects. The incidence of immune-related side effects in patients treated with the immune checkpoint inhibitor pembrolizumab is thought to be nearly 20% [2]. The pathogenesis of these side effects is varied and complex, and little remains known about what predisposes certain individuals as compared to others. Since current therapies are nonspecific, meaning they target tumor or benign parts of the body indiscriminately, sensitization against antigens possessed by both could play a role in the genesis of certain pathologies. Neurological side effects include reports of varying neuropathies, meningoencephalitis, myositis, and myasthenia gravis.

Classical myasthenia gravis is an autoimmune neurological disorder characterized by defective transmission at the neuromuscular junction. Most commonly, patients will develop autoantibodies against the acetylcholine receptors (AChRs), muscle-specific kinase (MuSK), and lipoprotein-related protein 4 (LPR4). The presence of these autoantibodies leads to the classic clinical manifestation of fatigable muscle weakness especially involving ocular, bulbar and respiratory muscles [3]. Interestingly enough, myasthenia gravis can also develop as a side effect to certain medication usage. While much isn’t known about the features or pathogenesis of this “de novo” disease, most cases involving the development of myasthenia gravis in the setting of PD-1 inhibitor use have observed acetylcholine receptor antibodies in around 66% of patients, with around 5.3% having anti-MuSK antibodies [4]. Additionally, if detected, titers are generally much lower than those seen in PD-1 inhibitor naive patients. Reports have indicated that patients with PD-1 inhibitor-induced myasthenia gravis frequently present with a rapidly progressive disease course featuring life-threatening bulbar or respiratory muscle involvement [4]. Less alarming symptoms, such as ptosis, diplopia, dysphagia, and dysarthria have similarly been observed [4]. 

As of now, no management protocol exists specifically for identification and management of patients with melanoma treated with pembrolizumab. In a literature review performed by Hajhossainlou et al., all documented cases of de novo MG prior to 2021 were identified [5]. Of the 36 cases identified, 10 cases were of patients diagnosed with melanoma; of these, only 7 were treated with pembrolizumab. Three of these cases developed severe respiratory failure and required subsequent mechanical ventilation, and ultimately 2/3 passed away. Therefore, there is a dearth of details surrounding management of patients with metastatic melanoma treated with pembrolizumab. The objective of this case report, then, is to highlight areas in management where clinical judgement had to be relied upon in the absence of clinical guidelines and call for recommendations surrounding identification of at-risk patient populations, standardized medical management, and hospital follow up.

## Case presentation

We present a 73-year-old male with a past medical history significant for melanoma, prostate cancer, hypothyroidism, and hypertension. He presented with clinical symptoms of myasthenia gravis and myositis shortly after beginning treatment with Pembrolizumab.

The patient was diagnosed with melanoma of the back approximately a year before presentation (Stage IIB) and underwent wide local excision. Additional workup including a sentinel lymph node biopsy and PET-CT scan was negative for any evidence of metastatic disease when adjuvant Pembrolizumab was started. He received his first two doses about two months prior to presentation. Both these doses were 200 mg in 0.9NaCL IV 118 mL infusion.

**Fig. 1:**
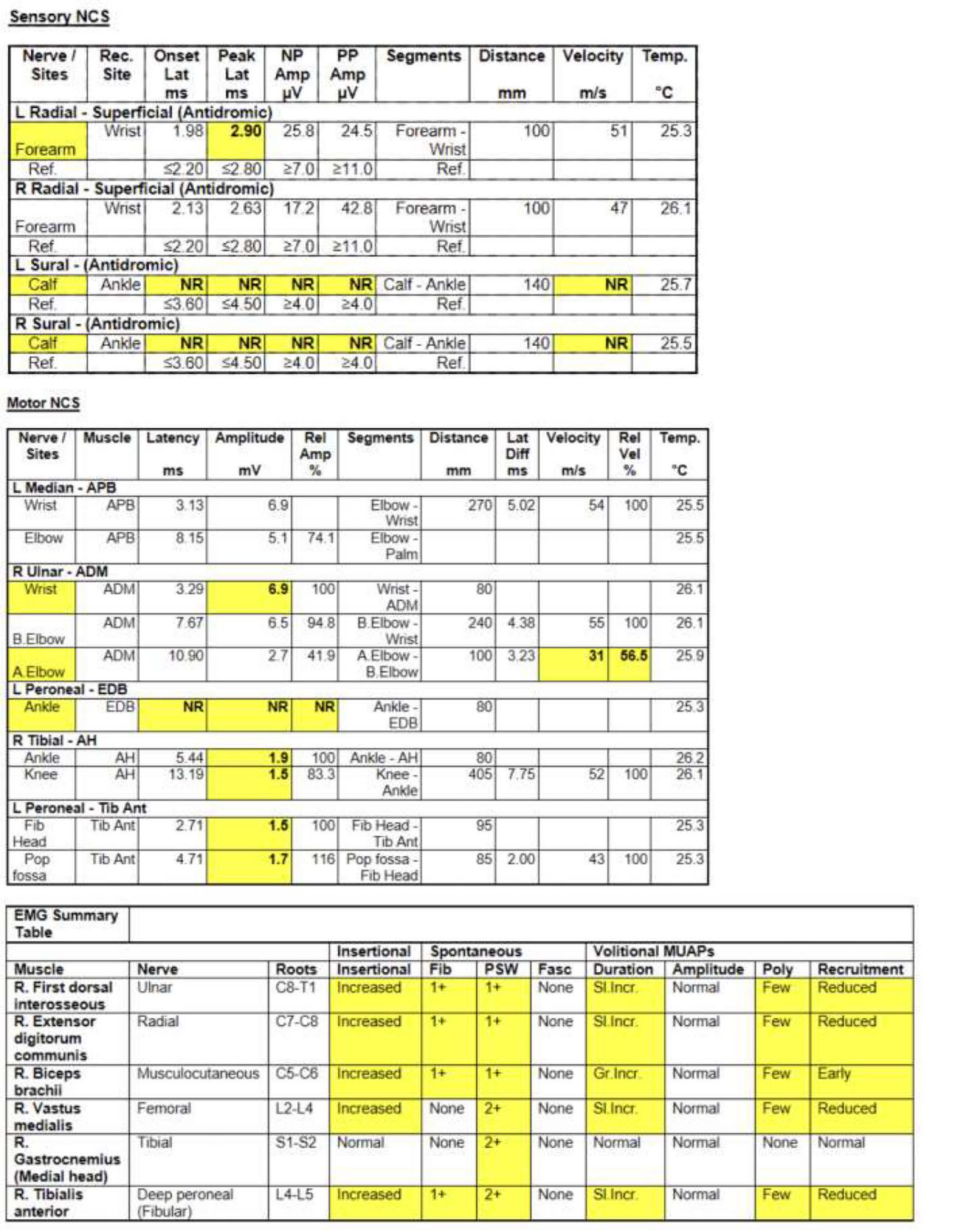
NCS/EMG summary data demonstrating electrophysiological evidence diffuse neuropathy and myopathy

Approximately two weeks after this second dose, he presented to the emergency department with progressive neck and back pain, dysphagia, hoarseness, generalized weakness, and urinary incontinence. Upon admission, he was noted to have creatinine kinase elevated to 2639 U/L (Normal 55–170 U/L), ALT elevated to 222 (Normal 0–61 U/L), and AST elevated to 200 (Normal 5–34 U/L). MRI of the brain and cervical spine did not show signs of metastasis but revealed a disc bulge at C3/C4 (shown in Fig. 1) causing canal stenosis. Neurological exam at this time was significant only for tenderness to palpation over the cervical neck. Both AChR and MuSK antibodies were negative, as was a paraneoplastic autoantibody panel. However, the possibility of MG secondary to Pembrolizumab could not be ruled out, and the decision was made to initiate empirical steroids. The patient was discharged home twelve days after admission with home health care and a steroid course.

One month following discharge, he was readmitted for failure to thrive in the setting of progressively worsening dysphagia, difficulty breathing, and generalized weakness. His exam was notable for neck extension weakness, right-sided ptosis, and inability to phonate. Within two days of admission, he developed hypoxic respiratory distress requiring intubation. On admission to the ICU, the patient was afebrile, tachycardic to 119 bpm, and hypotensive to 90/62 mmHg. His cardiac exam revealed no murmurs, rubs, or gallops in any of the cardiac windows. His labs were significant for CK 480 U/L (Normal 25-90U/L), ALT 168 U/L (Normal 0–61 U/L), AST 74 U/L (Normal 5–34 U/L), Tbili of 1.7 mg/dL (Normal 0.1-1.0 mg/dL), and WBC of 13,400 mm^3 (Normal 4,500 − 11,000 mm^3). Given that his clinical picture was consistent with myasthenic crisis, a 5-day course of high-dose steroids and plasma exchange was initiated. Following plasma exchange, his negative inspiratory force index remained low, but there was a significant improvement in his overall strength. Extubation was attempted two times given improving vital capacities, however the patient developed respiratory failure and required re-intubation. Pyridostigmine was started following the last session of plasma exchange for symptomatic treatment.

**Fig. 2:**
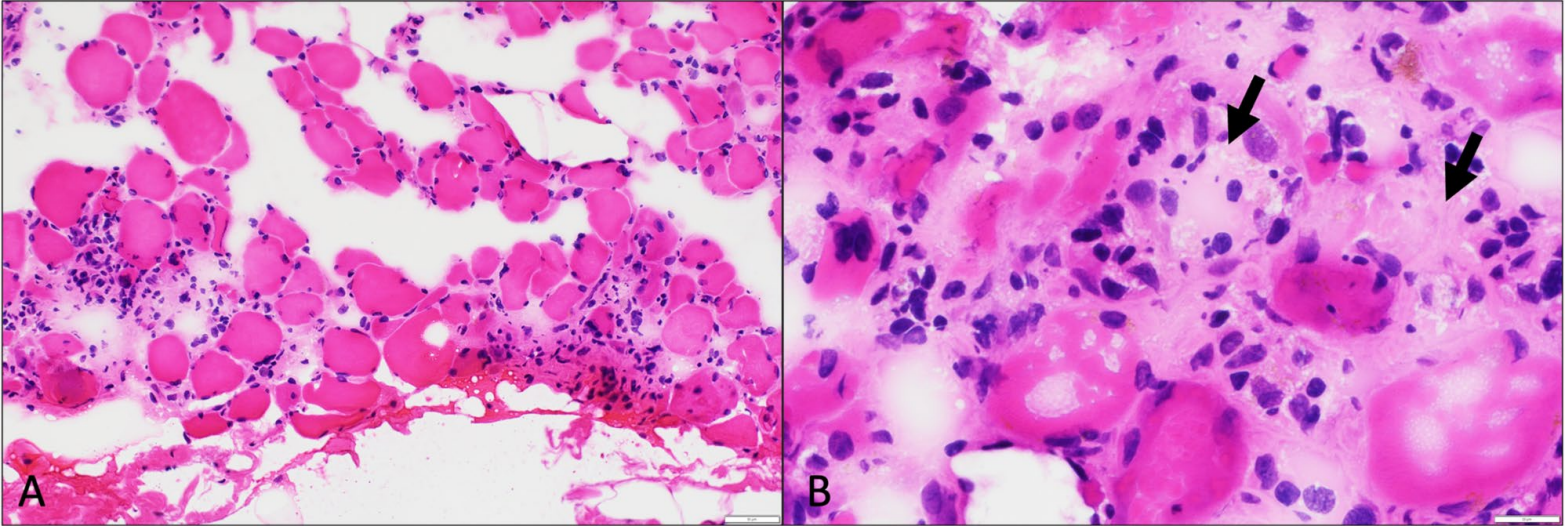
**A**. Muscle biopsy showing necrotizing myositis with associated inflammation (H&E, original magnification 20X). **B**. Necrotic fibers (black arrows) with surrounding lymphocytic and histiocytic inflammation in high power (H&E, original magnification 60X)

A repeat paraneoplastic panel, myositis panel, and LRP4 antibody workup were unrevealing during his second hospitalization. An NCS/EMG was done which demonstrated diffuse neuropathy with evidence of non-specific proximal myopathy (Fig. 1). A muscle biopsy of the left rectus femoris muscle showed myositis with necrotic fibers and inflammatory infiltrates, consistent with inflammatory myopathy and/or medication induced myositis (Fig. 2).

## Discussion

The mortality for autoimmune induced myasthenia gravis via PD-1 inhibitors is 29.8% while the mortality of classical myasthenia gravis is significantly lower at around 6% [6]. While this difference in mortality may be attributed to multiple factors such as age of onset, concomitant malignant and other cancer-related complications, checkpoint inhibitor-induced MG is often refractory to standard therapy for classical MG [4]. Therefore, we suggest that providers maintain a low threshold for suspecting neurological (namely, neuromuscular) derangements within the ICI-treated patient population.

Current treatment for classical myasthenia gravis includes acetylcholinesterase inhibitors, glucocorticoids, and thymectomy in patients with concurrent thymomas. Treatment for MG secondary to ICI autoimmune response is evolving. Once an MG flare has been observed in the setting of ICI use, the best course of action includes stopping usage of these inhibitors and beginning a course of low-dose corticosteroids or methylprednisolone (if namely inflammatory side effects persist) [1]. However, paradoxically it was also found that giving steroids in isolation may worsen the clinical picture. Instead, there is evidence that 95% of patients who are concomitantly treated with IVIG and PLEX improve to some degree [3]. It was also found that readministering the ICI after resolution of the MG symptoms with concurrent prednisone, IVIG, and pyridostigmine showed no recurrence of symptoms with partial or complete tumor response to the ICIs [7, 8]. 

We found that our patient, in addition to their de novo MG, also had myositis as evidenced by their elevated CK and biopsy results (Fig. 1). It is estimated that around 37% of all patients with de novo MG also have concomitant myositis [3]. While it has been suggested that the concomitant presentation of myositis or myocarditis in addition to de-novo MG may result in worsening clinical picture, further research is needed to understand the exact difference in outcomes [3]. 

It’s important to note that the presentation of de-novo MG has a more variable presentation than the standard MG. Recent literature has shown that de-novo MG correlates poorly with radiographic findings classically suggestive of MG such as thymoma or thymic hyperplasia [7, 9]. Other studies have suggested that some phenotypes might even be subclinical in nature; thus, in the setting of acute illness, some subtypes of de-novo MG may be missed. We suggest that providers strongly consider incorporating repetitive nerve simulation results in addition to clinical features when diagnosing de novo MG.

While clinical syndromes have been documented around the first two cycles of pembrolizumab, minimal research into possible primary prevention and exact medical management exists. Thus, it is unclear if early detection and intervention poses a therapeutic benefit. For example, Marco et al. discussed 6 patients treated with ICI and developed de novo MG; the median time to intubation of these patients was around 12–15 days, while our patient was intubated 2 days after symptom presentation [7]. Therefore, it is unclear when the “critical” or dangerous window is for de novo MG/when acute respiratory failure may present. Identifying this window early in hospital course might warrant early transfer to ICU and subsequently improve outcomes of this patient population. Additionally, the phenomenon of myocarditis/constellation of cardiac symptoms in patients with de novo MG has been well documented, but discussion/research needs to be done regarding serial management with echo and EKG- can this improve mortality in these patients? If so, when should this be initiated?

This case report details a patient diagnosed with ICI induced myasthenia gravis, the course of his care, and discusses the lack of a standard of care surrounding the treatment of de-novo MG.

One goal of this case report is to underscore the importance of early diagnosis of ICI induced MG/myositis while highlighting the absence of a standard of care for affected patients.

Many important questions surrounding diagnosis, prevention, and management of ICI induced MG and myositis remain answered. Further research examining how specific forms of prevention, diagnostic methods, and acute interventions influence outcomes and mortality would certainly be warranted and beneficial.

## Data Availability

Not applicable.

## Abbreviations

ICI: Immune Checkpoint Inhibitor
MG: Myasthenia Gravis
AChRs: Acetylcholine Receptors
MuSK: Muscle-Specific Kinase
LRP4: Lipoprotein-Related Protein 4
